# Effect of ruminal administration of soy sauce oil on rumen fermentation, milk production and blood parameters in dairy cows

**DOI:** 10.5713/ajas.19.0617

**Published:** 2019-12-24

**Authors:** Daiji Konno, Masanobu Takahashi, Ikuo Osaka, Takenori Orihashi, Kiyotaka Sakai, Kenji Sera, Yoshiaki Obara, Yasuo Kobayashi

**Affiliations:** 1 Graduate School of Agriculture, Hokkaido University, Sapporo, Hokkaido 0608589, Japan; 2 Dairy Research Center, Hokkaido Research Organization, Nakashibetsu, Hokkaido 086-1135, Japan; 3 Mito Research Center, Meiji Feed CO., LTD., Ibaraki, Ibaraki, 311-3123, Japan

**Keywords:** Dairy Cow, Fatty Acid, Milk, Rumen Fermentation, Soy Sauce Oil

## Abstract

**Objective:**

To evaluate soy sauce oil (a by-product of making whole soybean soy sauce) as a new dietary lipid source, a large amount of soy sauce oil was administered into the rumen of dairy cows.

**Methods:**

Four Holstein dairy cows fitted with rumen cannulae were used in a 56-day experiment. Ruminal administration of soy sauce oil (1 kg/d) was carried out for 42 days from day 8 to day 49 to monitor nutritional, physiological and production responses.

**Results:**

Dry matter intake and milk yield were not affected by soy sauce oil administration, whereas 4% fat-corrected milk yield and the percentage of milk fat decreased. Although ruminal concentration of total volatile fatty acids (VFA) and the proportion of individual VFA were partially affected by administration of soy sauce oil, values were within normal ranges, showing no apparent inhibition in rumen fermentation. Administration of soy sauce oil decreased the proportions of milk fatty acids with a carbon chain length of less than 18, and increased the proportions of stearic, oleic, vaccenic and conjugated linoleic acids. Conjugated linoleic acid content in milk became 5.9 to 8.8 times higher with soy sauce oil administration. Blood serum concentrations of non-esterified fatty acid, 3-hydroxybutyric acid, total cholesterol, free cholesterol, esterified cholesterol, triglyceride and phospholipid increased with administration of soy sauce oil, suggesting a higher energy status of the experimental cows.

**Conclusion:**

The results suggest that soy sauce oil could be a useful supplement to potentially improve milk functionality without adverse effects on ruminal fermentation and animal health. More detailed analysis is necessary to optimize the supplementation level of this new lipid source in feeding trials.

## INTRODUCTION

Conjugated linolenic acid (CLA), mainly *cis*-9 *trans*-11 CLA, has been extensively studied since the 1990s due to its anti-carcinogenic and anti-atherogenic functions [1,2], which can lead to positive effects on human health if adequately consumed. CLA is relatively abundant in food of ruminant origin [3] because it is generated within ruminant tissues such as the rumen and mammary gland. CLA is derived from linoleic acid via rumen microbial hydrogenation [4]. This can be outflowed from the rumen, absorbed in the lower tract and finally transferred into milk [5]. Also, CLA is derived from *trans*-11-octadecenoic acid (vaccenic acid), one of the further hydrogenated intermediates that can be converted to CLA in mammary gland [6]. As CLA in milk is primarily originated from dietary linoleic acid and its metabolites, the supply of such fatty acids is a key issue for production of milk with a higher proportion of CLA. It has been reported that CLA content in milk can be increased by feeding extruded soybean [7], roasted soybean [8] and pasturage [9], all of which are sources of linoleic acid. In the meantime, feeding oil often decreases feed intake by affecting rumen fermentation to lower fiber digestibility, intestinal hormones and hepatic fat oxidation [10–12]. In addition, milk fat decreases as the feeding amount of free or esterified fatty acid increases; therefore, dietary ether extract (EE) should be lower than 6% to 7% [13]. Thus, selection of lipid source and supplementation level are important.

Soy sauce oil, a by-product of making whole soybean soy sauce, is obtained by squeezing and separating a mash that has undergone a brewing process with yeast and lactic acid bacterium using whole soybeans, wheat and salt as raw materials. It might be expected that the CLA content of milk can be increased by feeding soy sauce oil to dairy cows since soy sauce oil is as rich in linoleic acid as soybean oil is. However, most soy sauce oil is currently used as a fuel alternative to heavy oil. If soy sauce oil is usable as a dietary lipid source for dairy cows, the application would be useful in terms of promotion of resource recycling, feed self-sufficiency rate and reduction of feed costs.

Preliminary evaluation of soy sauce oil as a dietary lipid for ruminants was carried out by Shibata et al [14]. They indicated that soy sauce oil has no negative effect on dry matter (DM) intake, milk yield and composition, while the proportion of CLA in milk fat increased with ruminal administration of soy sauce oil at 0.4 kg/d. Although that report pointed out the usefulness of soy sauce oil for dairy cows, the study was short (2 weeks) and provided limited information on the potential of this new lipid substrate. New feed materials, especially lipid substrates, should be evaluated in relation to feed intake since excess supply of dietary lipid can suppress DM intake, which can hamper livestock production performance. Therefore, responses to feed intake of soy sauce oil should also be assessed, especially to determine the upper limit of its supplementation.

The purpose of the present study was to clarify the effects of a large amount (1 kg/d) of soy sauce oil administration on feed intake, ruminal fermentation, blood parameters and dairy performance (milk production and composition) of cows over a long-term period. The soy sauce level of 1 kg/d was defined to temporarily set EE content of total feed at 10%. Our hypothesis was that this new lipid source is useful for improving milk quality in terms of functional fatty acid content without negatively affecting rumen fermentation and animal health. Although the amount of soy sauce oil administered is obviously not practical, to the best of our knowledge, this study is the first detailed evaluation of soy sauce oil with respect to what can be expected from this new lipid source.

## MATERIALS AND METHODS

### Animals and diets

All the research protocols regarding animal care followed the Guidelines for Animal Experimentation of the local independent administrative agency of the Hokkaido Research Organization.

Four primiparous Holstein dairy cows (587±93 kg body weight with 213±23 days in milk), fitted with 10-cm (internal diameter) soft plastic cannulae (Bar Diamond Inc., Parma, ID, USA), were used in a 56-day feeding experiment. They were fed a total mixed ration (TMR) consisting of 75.4% grass silage, 13.7% corn, 9.9% soybean meal, 0.7% calcium carbonate and 0.3% di-calcium phosphate on a DM basis (Table 1). For the adjustment of diet formulation, DM content of grass silage was determined weekly. TMR was given once daily (10.00 h) *ad libitum*, which was equivalent to 110% of the expected intake. Then, the amounts of diet offered and refused were recorded daily. Cows were housed in tie stalls with rubber mats bedded with sawdust. Fresh water was available at all times. Cows were milked twice daily (09:00 and 19:00) and milk production was recorded at each milking. Cows were weighed weekly during the feeding experiment.

For the treatment, soy sauce oil (Meiji Feed Co. Ltd., Tokyo, Japan) was directly administered via cannula into the rumen of each cow at a level of 1 kg/d during the period from day 8 to day 49. EE content in the total feed was about 10%. Soy sauce oil was administered twice daily (08:30 and 17:30) by equally dividing the daily allowance. Thus, the morning administration of oil was 1.5 h before TMR feeding. Soy sauce oil was not administered for the first 7 days and the last 7 days of the feeding experiment, which were regarded as pre- and post-administration periods.

### Sample collection

Milk was sampled on days 2, 5, 8 to 28, 30, 37, 44, 51, and 54 and divided into plastic vials for storage at −30°C for long-chain fatty acid analysis. Rumen and blood samples were obtained via the ruminal cannula and tail vein, respectively, at 08:00 on days 5, 6, 8, 9, 11, 13, 16, 19, 23, 26, 30, 37, 44, 51, and 54. In addition, on day 39 (week 6), rumen samples were taken at −0.5, 0, 0.5, 1, 2, 3, 4, 5, 6, 7, 8, and 9 h after the morning administration of soy sauce oil to determine diurnal changes in fermentation parameters.

Rumen sampling was done from the dorsal and ventral sacs of the rumen by hand, and rumen contents were mixed and then strained through four layers of surgical gauze to collect representative filtrate in a plastic vial. The pH of the filtrate was measured using an electrode (HM-50G, TOA-DKK, Tokyo, Japan), and then stored −30°C for further analysis. Blood samples were centrifuged to obtain serum, and stored at −30°C until analysis.

Feed samples including grass silage, corn and soybean meal were collected weekly and dried in a 60°C forced-air oven for 48 h for DM determination. The dried feed samples were ground and sieved through a 1-mm screen using a cutting mill and used for chemical analysis.

### Chemical and statistical analysis

Feed samples were analyzed for DM, crude protein (CP), EE, and neutral detergent fiber following standard procedures [15]. Milk samples were employed for the analysis of fat, protein, and lactose by infrared analysis (Milko-Scan FT120; Foss Electric, Hillerod, Denmark).

Frozen samples of rumen, milk and blood serum were thawed at 4°C and employed for the following analyses. Ammonia nitrogen was analyzed using the phenol hypochlorite method [16]. Volatile fatty acid (VFA) was analyzed a gas chromatograph (GC-14B; Shimadzu Co., Kyoto, Japan) using crotonic acid as an internal standard [17]. Fatty acid analysis was performed after conversion of fatty acids into fatty acid methyl esters (FAME), which were analyzed according to the method of Aii et al [18] with minor modifications. Briefly, after milk fat was extracted by hexane, FAME was prepared by the trans-methylation procedure according to Christie’s method [19]. Methyl esterified samples were analyzed using a gas chromatograph (GC-14B; Shimadzu Co., Japan) fitted with a flame ionization detector, using a silica capillary column (SP-2560, 100 m×0.25 mm (i.d.) with 0.20 μm film thickness; Spelco, Bellefonte, PA, USA). Biochemical analysis of blood serum was conducted using an automatic biochemical analyzer (SYNCHRON CX5, Beckman Coulter, Inc., Brea, CA, USA).

Data were averaged and are shown as representative values of each week (i.e. data shown for week 1 through week 8 are averages from all samples taken in the corresponding week). All the data were analyzed using JMP (SAS Institute Inc., Cary, NC, USA) as a paired t-test. A level of p<0.05 was defined as significant.

## RESULTS

Table 2 shows nutrient intake during the experimental period with or without soy sauce oil administration into the rumen. Total DM intake was not affected by soy sauce oil administration, and ranged from 12.9 to 14.0 kg/d. With administration of soy sauce oil, proportions of EE intake and total digestible nutrients intake in total DM intake increased, while the proportions of grass silage and CP intake in total DM intake decreased.

Milk yield and composition are shown in Table 3. Milk yield was unaffected by soy sauce oil administration, and ranged from 19.3 to 20.8 kg/d. However, fat-corrected milk yield and milk fat percentage decreased with soy sauce oil administration, and these persisted even after cessation of oil administration at week 8. Protein, lactose and solid-not-fat contents were not affected by ruminal administration of soy sauce oil.

Weekly changes of ruminal fermentation parameters including pH, ammonia N, and VFA in cows ruminally administered soy sauce oil are indicated in Table 4. Ruminal pH and concentration of ammonia were not affected by soy sauce oil. Although ruminal concentration of total VFA and proportions of acetate, propionate and butyrate were partially affected by soy sauce oil, the values were within normal ranges, showing no marked inhibition in rumen fermentation.

Diurnal changes of ruminal fermentation parameters on day 39 (week 6) in cows ruminally administered soy sauce oil are shown in Table 5. At 3 h after feeding and later (and at 1.5 h after administration of soy sauce oil and later), ruminal pH decreased and ammonia level increased. However, concentration of total VFA, molar proportions of acetate, propionate and butyrate were relatively stable.

Table 6 shows fatty acid composition and *cis*-9 *trans*-11 CLA content of milk. With administration of soy sauce oil, proportions of butyric (C4:0), caproic (C6:0), caprylic (C8:0), capric (C10:0), lauric (C12:0), myristic (C14:0), palmitic (C16:0), and margaric (C17:0) acids decreased, while those of stearic (C18:0), oleic (C18:1), *trans*-11 C18:1 (vaccenic) acids and *cis*-9 *trans*-11 CLA increased (Table 6). Other isomers such as *cis*-10 *trans*-12 CLA and *trans*-9 *cis*-11 CLA were not detected (CLA means *cis*-9 *trans*-11 CLA hereafter, unless stated otherwise). Increased proportions of vaccenic acid and CLA were consistent throughout soy sauce oil administration (weeks 2 to 7) and even after cessation of administration (week 8). CLA content in milk was elevated by soy sauce oil up to 212.3 mg/100 g of milk at week 5 of the experimental period.

Blood parameters are given in Table 7. Concentrations of non-esterified fatty acid (NEFA), 3-hydroxybutyric acid, total cholesterol, free cholesterol, esterified cholesterol, triglyceride, and phospholipid increased with administration of soy sauce oil, while those of glucose, aspartate aminotransferase, γ-glutamyl transpeptidase, inorganic phosphorus and magnesium were unaffected. Concentrations of blood urea nitrogen, total protein, cholesterol ester ratio and calcium showed stable values throughout the experimental period.

## DISCUSSION

The present study aimed to evaluate the influence of administration of a large amount of soy sauce oil on the nutrition and physiology of dairy cows, especially rumen fermentation. Although feeding dairy cows a high proportion of lipids causes a decrease in DM intake, it has little influence on ruminal fermentation [20]. The administration of soy sauce oil in the present study did not negatively influence feed intake (Table 2), even though EE proportion in the diet exceeded the recommended value (6% to 7%) [13]. The present results with no depressed feed intake, even with a diet having 10.2% to 11.0% EE, suggest that soy sauce oil could be an alternative lipid energy source in the ruminant diet.

Ruminal pH in the present study was within the optimal range (6.1 to 6.7) to ensure normal growth of major rumen microbes [21], and other ruminal parameters including ammonia and VFA were not greatly affected by the administration of 1 kg of soy sauce oil (Table 4). However, Shibata et al [14] reported that rumen fermentation was slightly inhibited by soy sauce oil at a lower level of administration (0.4 kg/d), as was observed for decreased VFA concentration. The reason for the different responses between the two studies is not clear, although the response in feed intake should be examined not by direct ruminal administration, but by a feeing study, and allowable supplementation levels should be proposed for the application of soy sauce oil. According to diurnal changes in rumen parameters at 6 weeks (fully adapted to soy sauce oil), administration of soy sauce oil did not seem to lead to abrupt changes in rumen fermentation (Table 5).

Generally, the decrease in milk fat can be mainly explained by the decrease of acetate production in the rumen [22] caused by lowered dietary proportion of roughage. Milk fat percentage was markedly decreased with soy sauce oil administration in the present study (Table 3) regardless of no apparent inhibition of ruminal acetate level (Table 4). Therefore, it is thought that the decreased milk fat is attributable to the bio-hydrogenation theory, as described below.

The bio-hydrogenation theory [23] is supported by the fact that particular intermediates via ruminal hydrogenation of linoleic acid are absorbed in the lower tract and inhibit milk fat synthesis in the mammary gland [23]. The *trans*-10 *cis*-10 CLA is one of such intermediates to inhibit milk fat synthesis [24]. In fact, milk fat yield decreases as the proportion of this CLA increases [25]. In addition, it has been indicated that *cis*-10 *trans*-12 CLA [26] and *trans-*9 *cis*-11 CLA [27] also inhibit milk fat synthesis. Soy sauce oil used in the present study is rich in C18 unsaturated fatty acids (83%) that can be converted to the above fatty acids to negatively influence milk fat synthesis in the mammary gland. This is a possible explanation for the milk fat decrease in the present study. However, all these possibilities are to be confirmed by biopsy experiments of liver, adipose tissue and mammary gland.

Feeding with a diet rich in polyunsaturated fatty acids as substrates for ruminal synthesis of vaccenic acid and CLA is a useful way to enhance the CLA content of milk. There have been reports that the CLA content of milk is doubled with feeding lactating dairy cows a diet containing 5% soybean oil [28], and more than doubled (2.3 times) by grazing in comparison with feeding TMR [9]. In this experiment using cows administered soy sauce oil rich in linoleic acid, the CLA proportion in milk fat and CLA content of milk increased 12.0- and 8.8-fold at maximum, respectively (Table 6). Such enrichment of CLA in milk is much higher than the values reported to date.

The NEFA, 3-hydroxybutyric acid (THB), cholesterol, triglyceride and phospholipid contents in blood serum were increased by ruminal administration of soy sauce oil (Table 7), as was observed for other oil/fat administration [29–31]. The increased blood lipids could be caused by soy sauce oil administration, but not by body fat mobilization because the body weight of experimental cows did not decrease in the present study (body weight ranged from 582 to 597 kg). These values did not return to the original (pre-treatment) level at 1 week after cessation of administration, as was observed for milk fat percentage and milk fatty acid profiles (Tables 3, 7), indicating a residual effect of soy sauce oil administration. Other blood parameters concerning hepatic function and mineral metabolism were fairly stable within normal levels throughout the experimental period. Therefore, it is thought that soy sauce oil does not adversely influence the health of dairy cows.

Although the present study represents the first detailed evaluation of the utilization of soy sauce oil as a new lipid source and further study is needed, we revealed that administration of soy sauce oil to dairy cows has the potential to increase the CLA content of milk. However, soy sauce oil could also reduce milk fat percentage, possibly due to absorption of hydrogenated fatty acid intermediates to inhibit milk fat synthesis. Obviously, the adequate dose level of soy sauce oil should be experimentally clarified not by direct ruminal administration as in the present study, but by more practical feeding studies.

## Figures and Tables

**Table 1:** Formulation and chemical composition of the experimental total mixed ration

Items	%
Ingredients (% dry matter)	
Grass silage	75.4
Corn	13.7
Soybean meal	9.9
Calcium carbonate	0.7
Dicalcium phosphate	0.3
Chemical composition (% dry matter)	
Crude protein	17.3
Ether extract	3.4
Neutral detergent fiber	47.3
Non fiber carnohydrate1)	22.5
Total digestible nutrients	64.5

1) Organic matter = (crude protein + ether extract + neutral detergent fiber).

**Table 2:** Effect of ruminal administration of soy sauce oil on nutrient intake in dairy cows

Items	Week of experiment1)								SE

	1	2	3	4	5	6	7	8	
Dry matter intake (kg)	13.9	14.0	12.9	13.0	13.9	14.0	13.7	13.6	0.21
Grass silage (dry matter intake, %)	75.4	70.0*	69.5*	69.5*	69.9*	70.0*	69.9*	75.4	0.45
Ether extract (dry matter intake, %)	3.3	10.2*	10.8*	11.0*	10.6*	10.4*	10.6*	3.5	0.57
Crude protein (dry matter intake, %)	17.0	15.8*	15.7*	15.9*	16.4*	16.4*	16.4*	17.7	0.11
Total digestible nutrient (dry matter intake, %)	64.5	75.0*	75.9*	75.8*	75.1*	75.0*	75.2*	64.5	0.87

SE, pooled standard error.

1) Administration period of soy sauce oil is from 2 to 7 week.

* Significantly different from the value in 1st week (p<0.05).

**Table 3:** Effect of ruminal administration of soy sauce oil on milk yield and composition in dairy cows

Items	Week of experiment1)								SE

	1	2	3	4	5	6	7	8	
Milk yield (kg/d)	20.8	20.8	19.7	19.3	20.4	20.0	19.9	19.5	0.43
4% fat corrected milk (kg/d)	21.4	21.7	18.9*	17.3*	17.3*	16.9*	17.1*	17.2*	0.54
Fat (%)	4.21	4.29	3.73*	3.31*	2.95*	2.97*	3.04*	3.22*	0.10
Protein (%)	3.23	3.12	3.12	3.08	2.99	3.00	2.98	3.00	0.04
Lactose (%)	4.31	4.34	4.30	4.28	4.27	4.31	4.33	4.33	0.02
Solid non fat (%)	8.46	8.45	8.43	8.36	8.23	8.28	8.30	8.31	0.05

SE, pooled standard error.

1) Administration period of soy sauce oil is from 2 to 7 week.

* Significantly different from the value in 1st week (p<0.05).

**Table 4:** Effect of ruminal administration of soy sauce oil on ruminal pH, ammonia nitrogen concentration, total volatile fatty acid concentration, and proportions of acetic acid, propionic acid and n-butyric acid

Items	Week of experiment1)								SE

	1	2	3	4	5	6	7	8	
pH	6.5	6.5	6.5	6.6	6.6	6.5	6.5	6.7	0.03
Ammonia-N (mg/dL)	7.8	7.7	8.2	6.7	9.0	9.6	12.2	8.6	0.46
Total VFA (mmol/dL)	9.8	9.3	10.9	8.1*	7.6	10.5	10.3	8.3*	0.28
Acetic acid (mol %)	69.2	67.8	65.5	68.0	70.6	68.1	67.4	71.0*	0.37
Propionic acid (mol %)	16.6	17.5	18.6	18.2*	18.1	18.2	18.9*	15.7	0.25
n-Butyric acid (mol %)	11.4	11.4	12.5	10.9	8.2*	10.4	10.2*	10.4	0.31

SE, pooled standard error; VFA, volatile fatty acid.

1) Administration period of soy sauce oil is from 2 to 7 week.

* Significantly different from the value in 1st week (p<0.05).

**Table 5:** Effect of ruminal administration of soy sauce oil on diurnal changes in ruminal pH, total volatile fatty acid concentration, and proportions of acetic acid, propionic acid and n-butyric acid

Items	Hours after administration of soy sauce oil1)												SE

	−0.5	0	0.5	1	2	3	4	5	6	7	8	9	
pH	6.7	6.6	6.6	6.6	6.4	6.1*	6.3*	6.2*	6.1*	6.2*	6.2*	6.2*	0.03
Ammonia-N (mg/dL)	7.9	9.6	10.0	8.7	14.4	20.2*	15.0*	14.6*	15.6*	13.4	9.6	12.3	0.64
Total VFA (mmol/dL)	8.5	8.8	8.8	8.4	10.0	11.1	10.2	10.2*	10.8*	10.7	10.0	10.3	0.20
Acetic acid (mol %)	68.2	67.5	67.1	67.4	66.2	66.0	67.6	67.8	66.8	67.3	68.0	66.5	0.17
Propionic acid (mol %)	17.4	17.9	18.0	17.8	18.8	18.4	16.9	16.3*	17.1	17.0	16.9	17.9	0.14
n-Butyric acid (mol %)	11.2	11.0	11.1	11.0	11.2	11.1	10.9	11.1	11.3	11.3	11.3	11.6	0.12

SE, pooled standard error; VFA, volatile fatty acid.

1) TMR is fed at 1.5 hours after ruminal administration of soy sauce oil.

* Significantly different from the value before administration (average of values at −0.5 and 0 hours) (p<0.05).

**Table 6:** Effect of ruminal administration of soy sauce oil on milk fatty acid composition in dairy cows

Items	Week of experiment1)								SE

	1	2	3	4	5	6	7	8	
Fatty acid composition (%)									
C4:0 (butyric acid)	4.1	4.3	3.6*	3.4	3.1	3.3*	3.4	3.6*	0.07
C6:0 (caproic acid)	2.0	1.7*	1.2*	1.1*	1.0*	1.0*	1.0*	1.4*	0.07
C8:0 (caprylic acid)	1.1	0.8*	0.5*	0.4*	0.4*	0.4*	0.4*	0.6*	0.05
C0:0 (capric acid)	2.3	1.6*	0.9*	0.8*	0.7*	0.7*	0.7*	1.2*	0.10
C12:0 (lauric acid)	2.7	1.9*	1.1*	1.1*	1.0*	1.1*	1.0*	1.6*	0.11
C14:0 (myristic acid)	11.1	8.1*	5.7*	5.6*	5.7*	6.1*	5.7*	8.6	0.00
C14:1 (myristoleic acid)	0.9	0.7*	0.4*	0.5*	0.6	0.6	0.5	0.9	0.04
C15:0 (pentadecylic acid)	1.2	1.0	0.9*	0.9*	1.0	1.1	1.0	1.2	0.02
C16:0 (palmitic acid)	33.0	25.9*	20.7*	20.9*	22.3*	21.7*	21.0*	26.6*	0.80
C16:1 (palmitoleic acid)	1.7	1.5*	1.6	1.9*	2.1*	1.8	1.9	1.9	0.04
C17:0 (margaric acid)	0.8	0.5*	0.4*	0.4*	0.4*	0.4*	0.4*	0.5*	0.03
C18:0 (stearic acid)	9.7	11.1	12.8*	11.1*	10.2	10.6	10.5	9.5	0.28
C18:1 (oleic acid)	20.9	21.2	25.0*	25.4*	24.2	25.4*	25.7*	26.2*	0.45
*trans*-11 C18:1 (trans-vaccenic acid)	1.4	9.9*	14.4*	14.7*	14.2*	13.3*	13.5*	5.4*	0.91
C18:2 (linoleic acid)	1.4	1.4	1.3	1.4	1.5	1.8	1.8	1.7*	0.06
*cis*-9 *trans*-11 CLA (conjugated linoleic acid)	0.6	3.4*	5.4*	6.3*	7.2*	6.1*	6.5*	3.1*	0.40
C18:3 (linolenic acid)	0.6	0.4*	0.3*	0.3*	0.4*	0.5	0.5	0.4*	0.02
Others (unidentified)	4.5	4.6	3.8*	3.8	4.0	4.1	4.5	5.6*	0.15
*cis*-9 *trans*-11 CLA content (mg/milk 100 g)	24.2	143.6*	200.0*	207.0*	212.3*	182.0*	198.4*	98.5*	11.97

SE, pooled standard error.

1) Administration period of soy sauce oil is from 2 to 7 week.

* Significantly different from the value in 1st week (p<0.05).

**Table 7:** Effect of ruminal administration of soy sauce oil on blood parameters in dairy cows

Items	Week of experiment1)								SE

	1	2	3	4	5	6	7	8	
Energy metabolism									
Glucose (mg/dL)	63	61	61	63	63	63	65	59	0.47
Non-esterified fatty acid (μEq/L)	142	201	264	239*	275*	184*	276	251	13.04
3-Hydroxybutyric acid (μmol/L)	616	493	443*	377	282*	494*	444	457	19.96
Protein metabolism									
Blood urea nitrogen (mg/dL)	14.2	14.1	13.9	12.6	14.4	16.6*	14.9	14.0	0.37
Total protein (g/dL)	6.2	6.6	6.8	6.1	5.7	5.7	5.6*	5.6	0.10
Albumin (g/dL)	2.7	2.8*	2.8*	2.6*	2.5*	2.6	2.7	2.7	0.02
Lipid metabolism									
Total cholesterol (mg/dL)	179	193*	234*	257*	269*	299*	317*	306*	9.44
Free cholesterol (mg/dL)	32	34	40*	46*	47*	50*	51*	49*	1.42
Esterified cholesterol (mg/dL)	147	159*	194*	212*	222*	249*	265*	257*	8.06
Cholesterol ester ratio (%)	82.5	82.5	82.9	82.2	82.6	83.3	83.8	84.1*	0.15
Triglyceride (mg/dL)	5.9	7.9	13.6*	10.5*	14.0*	16.0*	15.0*	13.0	0.81
Phospholipid (mg/dL)	215	246*	292*	314*	322*	348*	366*	328*	9.07
Hepatic function									
Aspartate aminotransferase (IU/L)	54	58	107	65	52	50	52	51	6.57
Gamma-glutamyl transpeptidase (IU/L)	24	25	26	25	24	24	24	25	0.58
Mineral metabolism									
Inorganic phosphorus (mg/dL)	5.7	5.9	5.8	5.5	5.1	6.1	6.4	5.9	0.10
Calcium (mg/dL)	10.1	10.5*	10.9*	10.4*	10.0	10.3	10.6	10.8*	0.07
Magnesium (mg/dL)	2.4	2.5	2.2	2.2	2.4	2.6	2.6	2.5	0.06

SE, pooled standard error.

1) Administration period of soy sauce oil is from 2 to 7 week.

* Significantly different from the value in 1st week (p<0.05).
